# Corrected QT Interval and Outcomes of Dialysis Patients with Symptomatic Peripheral Artery Disease: A Prospective Cohort Study

**DOI:** 10.3390/jcm13030654

**Published:** 2024-01-23

**Authors:** Shuang-Chin Lin, Hsin-Hua Chou, Ting-Yun Lin, Hsuan-Li Huang

**Affiliations:** 1 Division of Cardiology, Taipei Tzu Chi Hospital, Buddhist Tzu Chi Medical Foundation, 289, Jianguo Road, Xindian District, New Taipei City 231, Taiwan; xde10106@tzuchi.com.tw (S.-C.L.); n_1200449@tzuchi.com.tw (H.-H.C.); 2 School of Medicine, Tzu Chi University, Hualien City 970, Taiwan; 3 Division of Nephrology, Taipei Tzu Chi Hospital, Buddhist Tzu Chi Medical Foundation, 289, Jianguo Road, Xindian District, New Taipei City 231, Taiwan; 4 School of Post-Baccalaureate Chinese Medicine, Tzu Chi University, Hualien City 970, Taiwan

**Keywords:** dialysis, major adverse cardiovascular events, mortality, peripheral artery disease, QT interval

## Abstract

Background: Peripheral artery disease (PAD) is common and associated with a higher risk of cardiovascular morbidity and mortality in dialysis patients. A longer corrected QT (QTc) interval has been associated with adverse cardiovascular events and mortality in the general population and patients with end-stage kidney disease. However, little evidence is available on the predictive value of QTc in dialysis patients with PAD. Methods: We conducted a prospective cohort study of 356 dialysis patients with symptomatic PAD undergoing endovascular therapy. We performed the resting 12-lead electrocardiogram (ECG) at baseline. Cox regression analyses were used to assess the association of QTc with all-cause mortality and major adverse cardiovascular events (MACEs), defined as non-fatal myocardial infarction, non-fatal stroke, and cardiovascular death. Results: The mean age was 67.3 ± 11.5 years; 41.6% of participants were women. The median QTc was 471 (interquartile ranges 448–491) milliseconds (ms). During a median follow-up of 2.2 years, 188 (52.8%) patients died, and MACEs occurred in 119 (33.4%) patients. In multivariable-adjusted models, patients in tertile 3 of QTc levels had a significantly greater risk of all-cause mortality (hazard ratio [HR] 2.41, 95% confidence intervals [CI] 1.58–3.69) and MACEs (HR 1.90, 95% CI 1.15–3.13) than those in tertile 1. Similarly, each 10-ms increase in the baseline QTc predicted a higher risk of all-cause death (HR 1.15, 95% CI 1.09–1.21) and MACEs (HR 1.15, 95% CI 1.07–1.23). Conclusions: QTc prolongation was independently associated with adverse outcomes among dialysis patients with symptomatic PAD.

## 1. Introduction

Chronic kidney disease (CKD) has become a significant worldwide public health problem affecting >10% of the general population [1]. According to the 2021 United States Renal Data System (USRDS) annual report, the overall prevalent population of patients with end-stage kidney disease (ESKD) reached 809,103 in 2019, a 41.0% increase from 2009 [2]. Patients with ESKD are at increased risk of cardiovascular disease (CVD). Among them, peripheral arterial disease (PAD) was common and was present in 40.7% of patients receiving hemodialysis (HD), 28.1% of patients receiving peritoneal dialysis (PD), and 21.0% of patients with a kidney transplant [2]. Therapeutic strategies for symptomatic PAD include vasoactive medications, antiplatelets, and revascularization procedures such as endovascular therapy (EVT) or surgical bypass. In CKD patients with PAD, there was a 2.5-fold higher frequency of myocardial infarction during index hospitalization and a nearly 3-fold higher hospital mortality rate [3]. Moreover, according to USRDS, the leading cause of death among ESKD patients in 2020 was arrhythmia or sudden cardiac death (SCD), which accounted for 44.9% of deaths [2].

Simple, non-invasive 12-lead electrocardiography (ECG) can give valuable information to detect arrhythmia. Measuring QT interval on standard ECG is widely used to evaluate several clinical conditions, including electrolyte imbalance, medication-related cardiac toxicity, and inherited channelopathies [4,5]. The QT interval is defined as the time between the beginning of the Q wave and the end of the T wave, representing the duration of ventricular depolarization and repolarization [6]. Since QT interval depends on heart rate, corrected QT interval (QTc) is essential before assessing QT interval. The most commonly used for calculating QTc interval is the Bazett formula [7]. A prolongation of QTc interval is associated with increased cardiovascular (CV) mortality in patients with atrial fibrillation [8], coronary artery disease (CAD) [9], severe aortic stenosis [10], acute ischemic stroke [11], as well as PAD and diabetic foot [12]. In addition, a prolonged QTc was associated with all-cause mortality and SCD among patients on maintenance HD [13]. However, the role of QTc interval remained uncertain in dialysis patients with symptomatic PAD. Therefore, the present study aimed to examine the impact of QTc interval on all-cause mortality and long-term CV outcomes in dialysis patients with PAD undergoing endovascular procedures.

## 2. Methods

### 2.1. Study Design and Patients

This study used data from the Tzu-Chi Registry of ENDovascular Intervention for Peripheral Arterial Disease (TRENDPAD) cohort, a physician-initiated, prospective single-center observational registry of patients who had undergone EVT for symptomatic PAD. We enrolled 453 consecutive adult ESKD patients with maintenance dialysis who underwent successful EVT for PAD between July 2005 and December 2019. We defined the symptomatic PAD patients as disabling claudication or chronic limb-threatening ischemia manifested as rest pain, non-healing wounds, or gangrene. Exclusion criteria included patients with cancer, patients with a follow-up period of less than three months among survivors, patients with factors affecting the QTc measurement such as permanent pacemaker implantation, and current use of amiodarone, and dronedarone or tricyclic anti-depressants.

We reviewed all participants’ complete medical history and records at baseline. The diagnosis of CAD was ≥50% lumen stenosis in at least one major coronary artery by coronary angiography or a history of myocardial infarction. We defined congestive heart failure as the left ventricular (LV) ejection fraction < 40% using echocardiography or radionuclide ventriculography, routinely performed for patients within three months of index EVT. Atrial fibrillation was defined as any preceding history of atrial fibrillation. Cerebrovascular disease was defined as any previous history of stroke. We obtained the blood samples from patients after overnight fasting before the index EVT. All participants provided written informed consent. This study complied with the Declaration of Helsinki and was approved by the institutional review board of Taipei Tzu Chi Hospital (09-X-067).

### 2.2. Electrocardiogram

All participants had a standard 12-lead ECG at admission for EVT, using MAC 5500 machine 285 (GE Medical System, Milwaukee, WI, USA). To ensure that ECG parameters were not affected by the acute metabolic effects of hemodialysis, ECG was evaluated one day after routine hemodialysis. The QT interval was measured from the beginning of the earliest onset of the QRS complex to the end of T the wave, defined as where the tangent of the downslope intersects the baseline, in either lead II, V5, or V6, with the largest result recorded. We averaged the QT interval over ten seconds in patients presenting with atrial fibrillation. The Bazett correction (QTc = QT/√[RR interval]) was used to calculate the QTc interval in milliseconds (ms) [7].

### 2.3. Clinical Outcomes

The outcomes of interest were all-cause mortality and major adverse cardiovascular events (MACEs). A MACE was the composite endpoint of non-fatal myocardial infarction, non-fatal stroke, or CV mortality. We ascertained the mortality data, including the cause of death from reports via the family, review of hospital records, and retrieval of death certificates. CV deaths included SCD or death due to myocardial infarction, stroke, lethal arrhythmia, decompensated heart failure, valvular heart disease, and aortic or other vascular disorders. For all-cause mortality, patients were censored at the time of their last contact or at the end of follow up in June 2020. For MACEs, patients were censored at the time of their last contact, death unrelated to a CV event, or the end of follow up.

### 2.4. Statistical Analyses

We compared baseline characteristics based on the tertiles of QTc intervals. Continuous data with or without a normal distribution were expressed as means ± standard deviations or medians and interquartile ranges, which were compared using one-way ANOVA or the Kruskal–Wallis test, respectively. Categorical data were expressed as frequencies and percentages and compared using the Chi-squared test. Univariate and multivariate linear regression models assessed the association of clinically relevant variables with the QTc interval. Survival analysis was estimated by the Kaplan–Meier method and compared by log-rank test. We applied the Cox proportional hazards modeling to estimate the hazard ratios (HRs) of the clinical outcomes associated with QTc intervals as categorical (tertiles 2 and 3 relative to tertile 1) or continuous variables. The covariates in multivariate models included demographic factors (age, sex, and time on dialysis), CV comorbidities (diabetes mellitus, CAD, congestive heart failure, and atrial fibrillation), medications (renin–angiotensin–aldosterone system inhibitor, and statins), and laboratory data (potassium, albumin, and C-reactive protein (CRP)). To assess the reliability of the impact of QTc prolongation on each endpoint, we also examined the clinical outcomes of patients with or without QTc prolongation, defined as QTc > 450 ms in men and >460 ms in women [6]. A two-tailed *p* value of <0.05 was considered statistically significant. The software used for statistical analyses was Statistical Package of Social Sciences, version 20.0 (SPSS Inc., Chicago, IL, USA).

## 3. Results

### 3.1. Patient Characteristics

**Figure 1:**
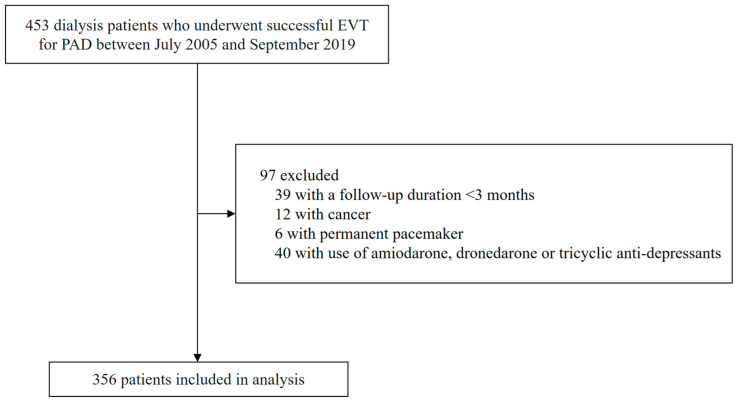
Patient flow diagram. EVT, endovascular therapy.

Of the 453 patients who were enrolled, 97 patients were excluded from further analyses (Figure 1). Among the 356 patients, the mean age of this cohort (*n* = 356) was 67.3 ± 11.5 years, 41.6% were women, and the median time on dialysis was 5.0 (2.0–8.0) years. We identified a significant burden of CV comorbidities in this population, of 86.2% with hypertension (*n* = 307), 83.4% with diabetes (*n* = 297), 55.9% with CAD (*n* = 199), 21.9% with congestive heart failure (*n* = 78), 18.0% with prior cerebrovascular accidents (*n* = 64), and 12.4% with atrial fibrillation (*n* = 44).

The median QTc interval was 471 (448–491) ms. Patients were categorized according to the tertiles of QTc (tertile 1: 369–458 ms; tertile 2: 459–484 ms; tertile 3: 485–568 ms) (Table 1). There was no significant difference in age, current smoking, dialysis method, body mass index, the prevalence of hypertension, diabetes, congestive heart failure, cerebrovascular disease, atrial fibrillation, use of CV medications, or serum potassium concentration across all patient groups. Compared with patients in tertile 1 and 2, those in tertile 3 had a shorter time on dialysis and were more likely to have CAD. In addition, they had lower levels of albumin, total cholesterol, high-density lipoprotein, and low-density lipoprotein but a significantly higher level of CRP.

Table 2 displays the association of clinically relevant variables with the QTc interval. In a univariate linear regression model, age, CAD, and the presence of congestive heart failure were positively correlated with QTc, whereas a longer time on dialysis and higher levels of albumin and low-density lipoprotein predicted a shorter QTc interval. In a multivariate model, only the presence of CAD predicted a longer QTc interval, while time on dialysis and serum albumin were negatively correlated with QTc interval.

### 3.2. Clinical Outcomes

During a median follow-up of 2.2 (0.9–4.4) years, we found 188 deaths (76 from CV death and 112 from non-CV death). The most common causes of non-CV deaths were sepsis (*n* = 67; 59.8%), pneumonia (*n* = 20; 17.9%), and malignancies (*n* = 8; 7.1%). One hundred and nineteen patients experienced a MACE, including non-fatal myocardial infarction (*n* = 37; 31.1%), non-fatal stroke (*n* = 19; 16.0%), and CV death (*n* = 63; 52.9%).

**Figure 2:**
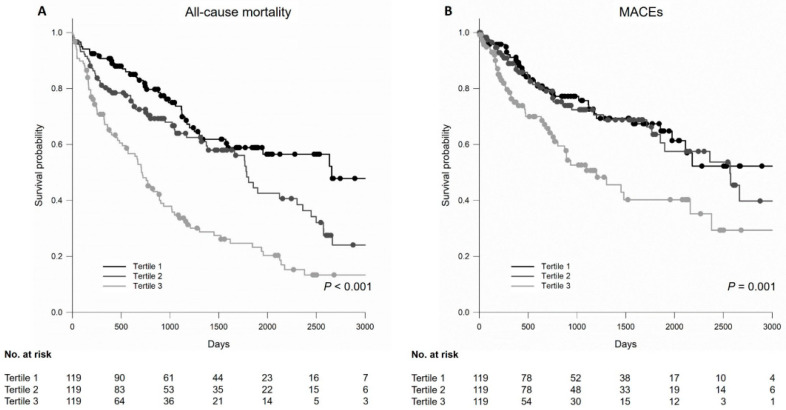
Kaplan–Meier survival curves for (**A**) all-cause mortality and (**B**) MACEs. MACE, major adverse cardiovascular event.

The Kaplan–Meier analysis examined the univariate association between the QTc tertiles and all-cause mortality (Figure 2A). Patients with higher tertiles of QTc showed an increased risk for death (*p* < 0.001). Next, we performed a Cox proportional hazards model to examine the mortality risk among the three groups (Table 3). Across the QTc tertiles, in unadjusted and adjusted models, patients in tertile 2 and 3 had a greater risk of morality than patients in tertile 1 (adjusted HR 1.64, 95% confidence intervals (CI) 1.06–2.55 and adjusted HR 2.41, 95% CI 1.58–3.69 for tertile 2 and tertile 3, respectively). Similarly, when treated as a continuous variable, every 1-ms higher QTc was significantly associated with death (adjusted HR 1.15, 95% CI 1.09–1.21).

As for MACEs, patients in the tertile 3 group showed the highest risk of MACEs (*p* = 0.001), whereas there was no difference between the tertile 2 and tertile 3 groups (Figure 2B). In the fully adjusted model, patients in tertile 3 were at a 1.90-fold (95% CI, 1.15–3.13) increase in the risk of developing a MACE (reference group: tertile 1). Likewise, in unadjusted and adjusted models, every 1-ms longer QTc was significantly associated with a 15% increased risk of MACEs (adjusted HR 1.15, 95% CI 1.07–1.23).

### 3.3. Sensitivity Analyses

Similar analyses were performed using the same multivariate models to assess the association between the clinical outcomes and the presence or absence of QTc prolongation, defined as QTc > 450 ms in men and >460 ms in women (Table 4). QTc prolongation was present in 70.2% of patients (*n* = 250).

We found that QTc prolongation predicted a higher risk of all-cause death (adjusted HR 2.41, 95% CI 1.59–3.64). We also found a significant association between QTc prolongation and an increased risk of MACEs in the unadjusted model (HR 1.68, 95% CI 1.10–2.55). Although the result became statistically nonsignificant in a fully adjusted model, the trend was consistent (adjusted HR 1.58, 95% CI 1.00–2.51).

## 4. Discussion

The main finding of this prospective study is that prolonged QTc interval constitutes an independent associated risk factor for all-cause mortality and MACEs in ESKD patients who had undergone the EVT for symptomatic PAD. A significant proportion of patients died (52.8%) or experienced MACEs (33.4%) during a median follow-up of 2.2 years. These results are partly contributed by various CV comorbidities in this population. Notably, the associations persisted even after multivariate adjustments, suggesting that a prolonged QTc is not simply a surrogate for more severe cardiac dysfunction but that additional mechanisms may underlie the association between QTc interval and poor prognosis.

Our findings aligned with prior studies showing the associations between QTc prolongation and traditional CV risk factors in patients with uremia, including aging and pre-existing CV diseases [14]. In addition, we also found serum albumin, a non-traditional CV risk factor, to be inversely associated with QTc intervals. Similar results have been reported. Wu et al. revealed that a lower serum albumin concentration was independently associated with QTc prolongation in a cohort of 1383 consecutive patients coexisting with CAD and CKD [15]. Shibata et al. also demonstrated serum albumin was negatively associated with QTc interval in a cohort of 224 dialysis patients [16]. Moreover, they found a longer QTc interval negatively associated with lower serum creatinine, worse nutritional status assessed by the Geriatric Nutritional Risk Index, and lesser muscle mass, suggesting the association between malnutrition and QTc prolongation. The mechanisms underlying nutritional status and QTc interval might be multifactorial. Malnourished dialysis patients with inadequate dietary intake may be more likely to develop hypokalemia, hypomagnesemia, and hypocalcemia [17]. These electrolyte disturbances can lead to a prolonged QTc interval. Indeed, in a large retrospective study of 9359 incident dialysis patients, those with a lower serum magnesium level were likelier to have lower serum albumin, potassium, and adjusted calcium [18].

Another possible factor linking hypoalbuminemia and prolonged QTc interval is systemic inflammation. Mounting evidence has strongly suggested that inflammation is a vital determinant of QTc prolongation in patients with autoimmune diseases [19]. Pisoni et al. have demonstrated that prolonged QTc was independently predicted by circulating interleukin (IL)-1β levels among 55 patients with connective tissue disorder [20]. Interestingly, the use of tocilizumab, an anti-IL-6 therapy, has been shown to be associated with a rapid QTc shortening in patients with rheumatoid arthritis, which was correlated with the decrease in both CRP and tumor necrosis factor (TNF)-α levels [21]. Although the mechanisms underlying inflammation-mediated QTc prolongation are not fully known, the key mediators might be inflammatory cytokines (particularly TNF-α, IL-6, IL-1β), which may affect myocardium either directly by modulating specific ion channels critically involved in cardiac action potential duration, and indirectly by increasing central nervous system sympathetic drive on the heart [19]. In this study, we found patients in tertile 3 of QTc have the highest level of CRP, although CRP did not independently correlate with QTc interval.

Interestingly, we found time on dialysis was inversely correlated with QTc interval, which contradicts previous studies. Matsumoto et al. reported that QTc intervals at 4 and 7 years after HD initiation were significantly prolonged compared to 1 year after starting HD [22]. The difference between previous findings and our results can be attributed to differences in population characteristics. CVD comorbidities were much higher in this cohort than in the prior study. Moreover, it is also likely that the inverse relationships observed in the present study were due to selection bias; that is, patients with longer QTc may have experienced earlier mortality than their counterparts with a shorter QTc interval.

Several studies support the impact of prolonged QT on adverse prognosis in patients with ESKD. Hage et al. evaluated 280 HD patients assessed for kidney transplantation, with a mean age of 53 ± 9 years; among them, 38% were female, 60% with diabetes, 21% with peripheral vascular disease, 63% with CAD, and 13% with cerebrovascular disease. They found that 39% of patients had a prolonged QTc, defined as QTc > 450 ms in men and >460 ms in women. In addition, QTc prolongation was an independent predictor of death (HR 1.01, 95% CI 1.00–1.01; *p* = 0.016) during 40 ± 28 months of follow-up, irrespective of age, gender, diabetes, myocardial infarction, presence and severity of CAD on angiography, LV hypertrophy, LV ejection fraction and multiple other variables [23]. Genovesi et al. also studied a case series of 122 HD patients [median age of 71.3 (62.9–76.6) years and 64.8% male], of which 27.1% with diabetes, 37.7% with CAD and 41.8% with dilated cardiomyopathy [24]. Using the same criteria as the studies of Hage et al. [23] and ours, 36% of patients had a prolonged QTc. They also demonstrated a prolonged QTc to be independently associated with a markedly increased risk of all-cause death (HR 2.16, 95% CI 1.20–3.91; *p* = 0.011) and SCD (HR 8.33, 95% CI 1.71–40.49; *p* = 0.009) during a median follow up of 3.9 years.

Our findings have important clinical implications. Currently, neither the United States nor European guidelines mention the role of the ECG in caring for patients with symptomatic PAD requiring the EVT [25,26]. We showed that QTc interval has a predictive role in adverse outcomes in a population with a high burden of CV comorbidities. Although the mechanisms remain unclear, this study reinforces the importance of the ECG as a simple tool for predicting long-term mortality and future CV events after EVT in dialysis patients with PAD. However, whether regular ECG measurements and timely intervention may change the prognosis in this population may need further study.

Our study had certain limitations that should be addressed. First, as is the case for any observational study, we could not establish the causality of the relationship between QTc and outcomes. However, we can illustrate the value of the QTc interval as an independent predictor in this population. Second, QTc intervals are dynamic over time. We only obtained a single time-point ECG for assessing QTc interval when HD patients received EVT. Third, this study did not collect important factors influencing the association between QTc and outcomes, such as serum electrolytes (calcium and magnesium), arterial blood gas, and various medications causing the prolonged QTc interval (except for amiodarone, dronedarone, and tricyclic anti-depressants), which could have contributed further to our understanding of the reasons for QTc prolongation and its association with prognosis in this population.

In conclusion, in this prospective cohort of 356 dialysis patients with symptomatic PAD undergoing EVT, a prolonged QTc interval was associated with an increased risk of all-cause death and MACEs even after accounting for demographic factors, comorbid conditions, and medication use. Given that QTc intervals are readily available through non-invasive ECG testing, we should incorporate measuring QTc into dialysis patients’ PAD care.

## Figures and Tables

**Table 1:** Baseline characteristics according to tertiles of QTc ^1^.

Characteristic	QTc			*p* Value
	Tertile 1 (*n* = 119)	Tertile 2 (*n* = 118)	Tertile 3 (*n* = 119)	
QTc (ms)	441 (428–448)	471 (465–477)	497 (491–513)	<0.001
Demographic data				
Age (year)	65.7 ± 11.8	67.4 ± 11.7	68.9 ± 10.8	0.096
Male sex, *n* (%)	42 (35.3%)	60 (50.8%)	46 (38.7%)	0.038
Current smoking, *n* (%)	31 (26.1%)	24 (20.3%)	26 (21.8%)	0.554
Dialysis method, *n* (%)				
Hemodialysis	112 (94.1%)	113 (95.8%)	109 (91.6%)	0.406
Peritoneal dialysis	7 (5.9%)	5 (4.2%)	10 (8.4%)	
Time on dialysis (year)	6.0 (2.5–10.0)	5.2 (2.4–8.0)	3.3 (1.6–7.5)	0.009
Body mass index (kg/m^2^)	23.8 ± 3.1	23.8 ± 3.7	24.5 ± 4.1	0.226
Comorbidity, *n* (%)				
Diabetes mellitus	102 (85.7%)	93 (78.8%)	102 (85.7%)	0.257
Hypertension	104 (87.4%)	102 (86.4%)	101 (84.9%)	0.850
Coronary artery disease	63 (52.9%)	57 (48.3%)	79 (66.4%)	0.014
Congestive heart failure	22 (18.5%)	22 (18.6%)	34 (28.6%)	0.098
Cerebrovascular disease	24 (20.2%)	20 (16.9%)	20 (16.8%)	0.747
Atrial fibrillation	8 (6.7%)	18 (15.3%)	18 (15.1%)	0.073
Medication, *n* (%)				
Antiplatelet	110 (95.7%)	113 (97.4%)	111 (95.7%)	0.722
Cilostazol	77 (67.0%)	71 (61.2%)	71 (61.2%)	0.579
RAASi	35 (30.4%)	38 (32.8%)	35 (30.2%)	0.896
CCB	45 (39.1%)	44 (37.9%)	34 (29.3%)	0.234
β-Blocker	58 (50.4%)	57 (49.1%)	56 (48.3%)	0.947
Statins	21 (18.3%)	33 (28.4%)	29 (25.0%)	0.182
Laboratory data				
Potassium (mmol/L)	4.0 (3.5–4.7)	3.8 (3.4–4.4)	3.8 (3.4–4.3)	0.055
Albumin (g/dL)	3.4 ± 0.7	3.6 ± 0.6	3.2 ± 0.7	<0.001
Total cholesterol (mg/dL)	153 (131–173)	159 (138–196)	141 (124–168)	0.002
Triglycerides (mg/dL)	122 (90–165)	127 (88–191)	133 (94–194)	0.338
HDL (mg/dL)	36 (27–44)	39 (31–48)	34 (26–42)	0.007
LDL (mg/dL)	90 (70–109)	88 (72–114)	80 (65–96)	0.013
Hematocrit (%)	31.9 ± 5.1	32.5 ± 5.8	31.3 ± 5.0	0.248
Creatinine (mg/dL)	7.0 ± 2.7	6.7 ± 2.4	6.7 ± 2.7	0.652
HbA1c (%)	7.0 (6.0–8.5)	6.7 (5.7–7.8)	6.8 (5.9–8.0)	0.283
CRP (mg/dL)	2.0 (0.4–8.2)	1.6 (0.6–6.4)	3.4 (1.1–11.5)	0.006

CCB, calcium channel blocker; CRP, C-reactive protein; HDL, high-density lipoprotein; LDL, low-density lipoprotein; RAASi, renin–angiotensin–aldosterone system inhibitor. ^1^ Tertile 1: 369–458 ms; Tertile 2: 459–484 ms; Tertile 3: 485–568 ms.

**Table 2:** Baseline characteristics as determinants of the QTc interval.

Variables	Univariate		Multivariate ^1^	
	β (SE)	*p* Value	β (SE)	*p* Value
Age (year)	0.31 (0.14)	0.025	–	–
Male sex	1.00 (3.28)	0.761	–	–
Time on dialysis (year)	−0.76 (0.32)	0.019	−1.03 (0.35)	0.004
BMI (kg/m^2^)	0.45 (0.44)	0.311	–	–
Diabetes mellitus	0.83 (4.34)	0.848	–	–
Hypertension	−1.85 (4.69)	0.694	–	–
Coronary artery disease	7.12 (3.23)	0.028	7.62 (3.4)	0.025
Congestive heart failure	7.83 (3.88)	0.044	–	–
Cerebrovascular disease	−5.41 (4.20)	0.198	–	–
Atrial fibrillation	6.74 (4.89)	0.169	–	–
Potassium (mmol/L)	−4.10 (4.69)	0.050	–	–
Albumin (g/dL)	−5.85 (2.45)	0.017	−7.76 (2.63)	0.003
Total cholesterol (mg/dL)	−0.06 (0.04)	0.135	–	–
HDL (mg/dL)	−0.10 (0.14)	0.442	–	–
LDL (mg/dL)	−0.12 (0.06)	0.028	–	–
CRP (mg/dL)	0.37 (0.23)	0.116	–	–

BMI, body mass index; CRP, C-reactive protein; HDL, high-density lipoprotein; LDL, low-density lipoprotein. ^1^ Stepwise method using all covariates in univariate analyses (adjusted R^2^ = 0.07).

**Table 3:** Associations between QTc and clinical outcomes.

Outcome	Unadjusted		Model 1		Model 2	
	HR (95% CI)	*p* Value	HR (95% CI)	*p* Value	HR (95% CI)	*p* Value
All-cause mortality						
QTc, continuous						
Per 10-ms increment	1.17 (1.12–1.23)	<0.001	1.16 (1.11–1.22)	<0.001	1.15 (1.09–1.21)	<0.001
QTc, categorical ^1^						
Tertile 1	Reference		Reference		Reference	
Tertile 2	1.63 (1.09–2.42)	0.016	1.58 (1.05–2.38)	0.030	1.64 (1.06–2.55)	0.028
Tertile 3	3.14 (2.16–4.56)	<0.001	2.84 (1.90–4.24)	<0.001	2.41 (1.58–3.69)	<0.001
MACEs						
QTc, continuous						
Per 10-ms increment	1.14 (1.07–1.32)	<0.001	1.13 (1.06–1.20)	<0.001	1.15 (1.07–1.23)	<0.001
QTc, categorical ^1^						
Tertile 1	Reference		Reference		Reference	
Tertile 2	1.20 (0.75–1.91)	0.440	1.16 (0.72–1.87)	0.551	1.19 (0.71–1.98)	0.516
Tertile 3	2.12 (1.36–3.32)	0.001	1.79 (1.11–2.86)	0.016	1.90 (1.15–3.13)	0.012

MACE, major adverse cardiovascular event. Model 1 is adjusted for age, sex, time on dialysis, diabetes mellitus, coronary artery disease, congestive heart failure, and atrial fibrillation. Model 2 is adjusted for covariates in Model 1, renin–angiotensin–aldosterone system inhibitor, statins, potassium, albumin, and C-reactive protein. ^1^ Tertile 1: 369–458 ms; Tertile 2: 459–484 ms; Tertile 3: 485–568 ms.

**Table 4:** Associations between prolonged QTc and clinical outcomes.

Outcome	Unadjusted		Model 1		Model 2	
	HR (95% CI)	*p* Value	HR (95% CI)	*p* Value	HR (95% CI)	*p* Value
All-cause mortality						
QTc ^1^						
Normal	Reference		Reference		Reference	
Prolonged	2.44 (1.68–3.54)	<0.001	2.39 (1.62–3.52)	<0.001	2.41 (1.59–3.64	<0.001
MACEs						
QTc ^1^						
Normal	Reference		Reference		Reference	
Prolonged	1.68 (1.10–2.55)	0.016	1.62 (1.04–2.53)	0.035	1.58 (1.00–2.51)	0.051

MACE, major adverse cardiovascular event. Model 1 is adjusted for age, sex, time on dialysis, diabetes mellitus, coronary artery disease, congestive heart failure, and atrial fibrillation. Model 2 is adjusted for covariates in Model 1, renin–angiotensin–aldosterone system inhibitor, statins, potassium, albumin, and C-reactive protein. ^1^ Prolonged: >450 ms in men and >460 ms in women.

## Data Availability

The data presented in this study are available on request from the corresponding author. The data are not publicly available due to privacy considerations.
